# Magnetic Garments Promote Parasympathetic Dominance and Improve Sleep Quality in Male Long-Distance Runners Following a 30 km Run

**DOI:** 10.3390/s24216820

**Published:** 2024-10-23

**Authors:** Ayaka Nobue, Kanae Sano, Masaki Ishikawa

**Affiliations:** 1 Faculty of Medical Science Technology, Morinomiya University of Medical Sciences, Osaka 559-8611, Japan; ayaka_nobue@morinomiya-u.ac.jp; 2 Faculty of Health and Well-Being, Kansai University, Osaka 590-8515, Japan; kanae_s@kansai-u.ac.jp; 3 Graduate School of Sport and Exercise Sciences, Osaka University of Health and Sport Sciences, Osaka 590-0459, Japan

**Keywords:** magnetism, sleep quality, autonomic nervous system, recovery, long-distance runners

## Abstract

This study aimed to investigate the effects of high-intensity running on the autonomic nervous system and sleep quality of male long-distance runners and to examine the impact of wearing magnetic garments on these parameters. Fifteen highly trained male collegiate long-distance runners participated in a randomized, double-blind crossover study. Participants completed two 30 km runs (30k-RUN) during a 10-day training camp. After each run, they wore either magnetic (MAG) or non-magnetic control (CTRL) garments. Sleep quality and heart rate variability (HRV) were assessed using a wrist-worn device before and after each 30k-RUN. Wearing MAG garments post-30k-RUN resulted in significantly longer deep sleep duration compared to CTRL. HRV analysis revealed that the MAG condition led to a significantly higher root mean square of successive RR interval differences and high-frequency power, indicating enhanced parasympathetic activity. The low-frequency to high-frequency ratio was significantly lower in MAG than in CTRL. Perceived recovery scores were significantly higher in MAG than in CTRL. The findings of this study suggest that wearing magnetic garments following high-intensity endurance running may promote parasympathetic dominance and improve sleep quality in male long-distance runners. These findings indicate that magnetic garments may be a practical method for enhancing recovery in athletes following intense training.

## 1. Introduction

Strategies for effective fatigue recovery and its monitoring in competitive sports have become increasingly pertinent for enhancing athletic performance. Non-pharmacological mechanical interventions, such as cryotherapy and stretching, have been investigated for their efficacy in recovery [1,2,3]. Moreover, sleep is considered a crucial factor affecting fatigue recovery and performance. Consequently, sleep deprivation may adversely impact physiological responses to races or exercises, potentially impeding physiological and psychological recovery [4]. However, elite athletes who engage in high-intensity [5] and high-volume [6] training often experience compromised sleep quality [7].

Intervention targeting the autonomic nervous system, which modulates sleep quality, has garnered significant attention. Endurance athletes, for instance, typically undergo prolonged training sessions, high training volumes, and extended periods of elevated stress. When their training intensity surpasses a certain threshold, sympathetic nervous system dominance is ensured, resulting in decreased sleep quality and diminished parasympathetic activity [8], which may impede recovery from fatigue [9]. Therefore, approaches that promote parasympathetic dominance following a race or high-intensity training are essential for improving sleep quality and facilitating recovery from fatigue.

Research suggests that exposure to magnets can enhance cellular repair processes and improve blood flow in both animals [10,11] and humans [12,13]. These effects may potentially facilitate fatigue recovery. However, there is limited research on the quantitative and qualitative effects of magnetic field exposure on fatigue recovery and sleep in competitive athletes. Magnetism may modulate the autonomic nervous system and sleep quality, potentially augmenting athletes’ recovery following intense training and competitions. The relationship between magnetic exposure during sleep and various aspects of sleep quality remains poorly elucidated, particularly in athletes undergoing high-intensity endurance training.

The purpose of this study was, therefore, to investigate the immediate effects of high-intensity and high-volume running training on the autonomic nervous system and sleep quality of long-distance runners and to examine the impact of wearing magnetic garments on these parameters following such training. We hypothesized that long-distance runners would exhibit sympathetic nervous system dominance and diminished sleep quality after high-stress running training. Additionally, we postulated that wearing magnetic garments would promote parasympathetic dominance and enhance sleep quality following running training.

## 2. Methods

### 2.1. Participants

Fifteen highly trained male collegiate long-distance runners participated in this study. Their mean age was 19.7 ± 1.0 years; mean height was 170.1 ± 3.7 cm; mean body mass was 54.0 ± 3.2 kg. Their best 5000 m run time was 14′06″8 ± 14″0 (corresponding to a monthly training volume of at least 700 km). All runners were members of the same collegiate team and had comparable levels of competitiveness, which standardized their training regimens. This study was conducted during a 10-day training camp. Throughout the training camp, participants were instructed to adhere to their prescribed training program, and their sleep–wake schedules were regulated. Dietary intake was standardized, with all participants consuming identical meals each day. The same dietary pattern was maintained for both halves of the training camp. The exclusion of female athletes was due to the difficulty in accounting for menstrual cycle timing and controlling training programs within the limited study period. Prior to enrollment, participants underwent screening for medication use (those regularly using medications known to alter heart rate variability (HRV) were excluded), alcohol consumption, smoking habits, and chronic diseases or illnesses. Before participation, all runners provided written informed consent after receiving comprehensive information about the study’s purpose and associated risks. This study was approved by the Ethics Committee of Morinomiya Medical University in Japan (authorization number 2022-147).

### 2.2. Experimental Protocols

Following a 5 km practice run on the preceding day, participants completed a 30 km run (30k-RUN), which was considered a high-intensity training session relative to their current fitness level. Participants wore standardized cotton attire the night before measurements and were randomly assigned to wear polyester garments (RESNO, Colantotte Co. Ltd. Osaka, Japan) either with or without magnets (MAG and CTRL conditions, respectively) following the 30k-RUN on two separate occasions. The magnetic garment contained magnets with a strength of 180 millitesla (mT) per magnet, with 10 magnets arranged on the back of the shirt and 6 magnets on the waist of the long pants. Participants wore the assigned garments for the entire sleep duration following the 30k-RUN. The CTRL garments were identical but lacked magnetic properties. Sleep parameters (duration, deep/light sleep, REM sleep, wakefulness), HRV, and skin temperature were monitored using a wrist-worn heart rate sensor.

**Figure 1:**
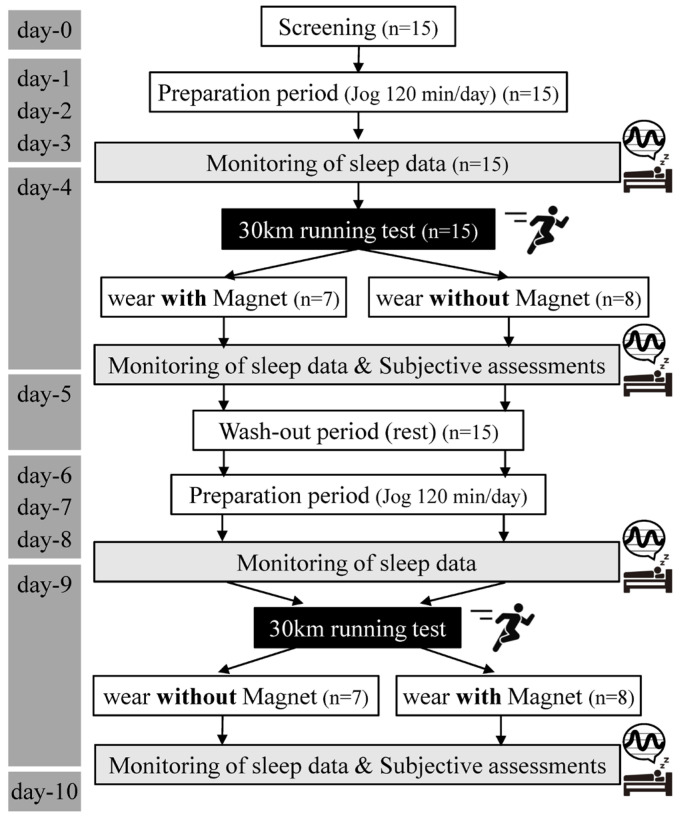
Schematic representation of the randomized, double-blind, crossover study design. Following a preparation period, participants were randomly allocated to either the magnetic (MAG) or non-magnetic control (CTRL) garment condition. Sleep parameters were assessed during the nights preceding and following the 30k-RUN. After a washout day period, participants crossed over to the alternate condition, and identical measurements were conducted.

Participants completed two 30k-RUN trials separated by 5 days during the 10-day training camp. This 30k-RUN protocol involved progressively increasing running speeds every 5 km: 16.4; 17.1; 18.0; 18.9; 19.5; and 20.0 km h^−1^ (Figure 1). Sleep data and HRV were measured during bedtime on two high-intensity endurance training days and the day preceding each trial. Upon awakening, perceived recovery and deep sleep scores were collected. This study employed a randomized, double-blind, crossover design, with all runners wearing either MAG or CTRL garments at bedtime following high-intensity training. Training sessions throughout the camp were standardized for all participants.

### 2.3. Procedures

Participants wore the wristband monitoring device continuously from the first day of the training camp and were habituated to sleep data acquisition (i.e., sleep time, sleep stage, and HRV) during the initial half of the camp. Subjective assessments of recovery and sleep were obtained from each participant within 30 min of waking following high-intensity training sessions. A wearable activity wristband (Sense, Fitbit Inc. San Francisco, CA, USA) and the Fitbit application program interface (API) were utilized to measure sleep parameters and HRV during the post-training sleep period. Sleep data comprised wake, light, deep, and REM sleep duration, as collected by the Fitbit device. For HRV analysis, this study employed the logarithm of the square root of the mean sum of squares of differences between adjacent regular R-R intervals (RMSSD), a recommended measure for monitoring athletes’ training status [14]. The use of RMSSD was justified by its sensitivity to changes in autonomic regulation and its practicality for assessing recovery status. Frequency domain activity was evaluated by deriving high frequency (HF) power (0.15–0.4 Hz) and low frequency (LF) power (0.04–0.15 Hz) from the wristbands, with subsequent calculation of the LF: HF ratio. Previous studies have demonstrated the consistency of Fitbit-derived sleep data and HRV measurements with polygraphs and ECGs, reporting high accuracy with a macro F1 score of 0.5564 for deep-sleep stage classification and up to 80% accuracy for total sleep time and sleep efficiency [15,16,17].

Perceived recovery and deep sleep quality were assessed using a 10-point Likert scale questionnaire, where “1” represented “very, very poor” and “10” represented “very, very good”. This subjective assessment was conducted within 30 min of waking in the morning following high-intensity training sessions.

### 2.4. Statistical Analyses

Results are presented as mean ± standard deviation. The effect of garment type and pre-post 30k-RUN on total and stage-specific sleep durations, as well as HRV parameters, were assessed using two-way repeated-measures ANOVA (rmANOVA) (condition [2: MAG and CTRL] × pre-post [2: pre- and post-30k-RUN]). Prior to rmANOVA analyses, assumptions of data normality and homoscedasticity were verified using Kolmogorov–Smirnov and Levene’s tests, respectively. When rmANOVA results were statistically significant, Tukey’s post hoc tests were employed to identify specific group differences.

For relative values of autonomic nervous system (ANS) parameters (RMSSD, HF, LF/HF, HF/(LF + HF)), normality was assessed using the Shapiro–Wilk test. Subsequently, two-tailed *t*-tests were conducted to compare differences between MAG and CTRL conditions before and after the run. Subjective recovery and deep sleep score comparisons were also analyzed using two-tailed *t*-tests.

All statistical analyses were performed using Jamovi software (Version 2.3.28.0, The Jamovi Project 2024, Sydney, Australia). A post-hoc power analysis was conducted using G*Power software (version 3.1.9.7) to validate the study’s findings. For two-way rmANOVA, the power to detect a medium effect size (Cohen’s *f* = 0.25) with the given sample size was 0.43 at an alpha level of 0.05. For the two-tailed t-test, the power to detect a medium effect size (Cohen’s *d* = 0.50) was 0.44 at an alpha level of 0.05. Given the limited statistical power, results should be interpreted cautiously. The significance level was set at *p* < 0.05. Effect sizes were interpreted as follows: for *t*-tests, small (*d* = 0.2), medium (d = 0.5), and large (*d* = 0.8) [18]; for rmANOVA, small (*η_p_*^2^ = 0.01), medium (*η_p_*^2^ = 0.06), and large (*η_p_*^2^ = 0.14).

## 3. Results

### 3.1. Sleep Analyses

**Figure 2:**
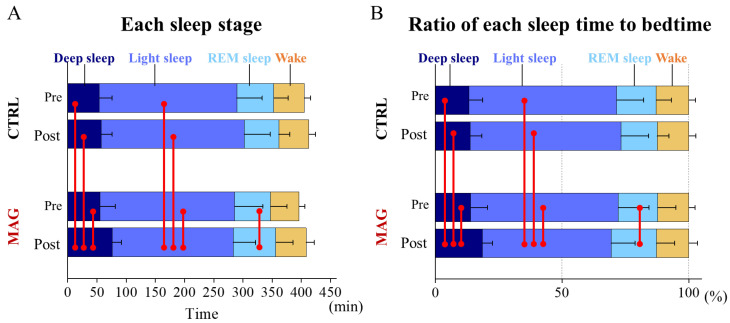
Absolute sleep stage durations (**A**) and relative proportions of total sleep time (**B**) before and after 30k-RUN in CTRL and MAG conditions. (**A**) Absolute sleep stage durations are presented as mean ± standard deviation. (**B**) Relative sleep stage durations are expressed as percentages of total sleep time. Red lines indicate statistically significant differences between conditions (*p* < 0.05).

This study compared sleep patterns between participants wearing MAG and CTRL garments before and after the 30k-RUN. Total sleep time shows no significant differences based on garment conditions (*F*_(1,14)_ = 0.644, *p* = 0.436, *η_p_*^2^ = 0.044) or pre-post conditions (*F*_(1,14)_ = 1.110, *p* = 0.310, *η_p_*^2^ = 0.073), with no interaction between these factors (*F*_(1,14)_ = 0.001, *p* = 0.988, *η_p_*^2^ < 0.01) (Figure 2A). Deep sleep duration exhibited a significant interaction (*F*_(1,14)_ = 4.654, *p* = 0.049, *η_p_*^2^ = 0.249) and a main effect for garment condition (*F*_(1,14)_ = 9.331, *p* = 0.009, *η_p_*^2^ = 0.400). Post-hoc Tukey’s tests revealed significantly longer deep sleep duration in the MAG-post condition compared to MAG-pre (*p* = 0.026), CTRL-pre (*p* = 0.005) and CTRL-post (*p* = 0.008). Light sleep duration demonstrated a main effect for garment conditions (*F*_(1,14)_ = 7.340, *p* = 0.017, *η_p_*^2^ = 0.344) and a significant interaction (*F*_(1,14)_ = 6.477, *p* = 0.023, *η_p_*^2^ = 0.316). Post-hoc Tukey’s tests showed significantly shorter light sleep duration in the MAG-post condition compared to MAG-pre (*p* = 0.039), CTRL-pre (*p* = 0.029), and CTRL-post (*p* = 0.001). For REM sleep, two-way rmANOVA revealed no significant main effects on garment condition (*F*_(1,14)_ = 0.896, *p* = 0.360, *η_p_*^2^ = 0.060) or pre-post conditions (*F*_(1,14)_ = 0.728, *p* = 0.408, *η_p_*^2^ = 0.049), but revealed a significant interaction between these factors (*F*_(1,14)_ = 4.902, *p* = 0.044, *η_p_*^2^ = 0.259). Post-hoc analysis indicated significantly longer REM sleep duration in MAG-post compared to MAG-pre (*p* = 0.049). The wake stage showed no significant interaction (*F*_(1,14)_ = 0.389, *p* = 0.543, *η_p_*^2^ = 0.027) or main effects for garment condition (*F*_(1,14)_ = 0.388, *p* = 0.543, *η_p_*^2^ = 0.027) and pre-post conditions (*F*_(1,14)_ = 0.162, *p* = 0.693, *η_p_*^2^ = 0.011). Relative sleep stage durations exhibited similar trends to absolute values (Figure 2B).

### 3.2. Autonomic Nervous System Parameters

**Figure 3:**
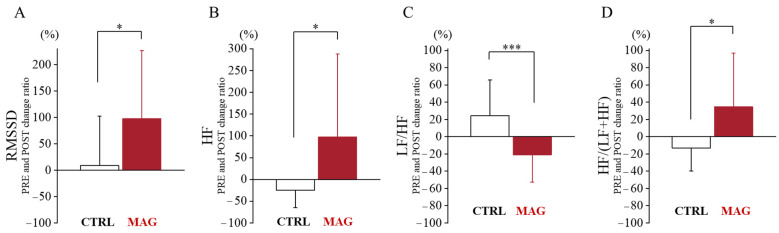
Relative changes in ANS parameters following 30k-RUN in CTRL and MAG conditions. (**A**) RMSSD (Root Mean Square of Successive RR interval Differences) ratio: (**B**) HF (High-Frquency) power ratio: (**C**) LF/HF (Low-Frequency/High-Frequency) ratio: (**D**) HF/(LF+HF) ratio: A normalized measure of parasympathetic activity. * and *** show significant differences between CTRL and MAG conditions (*p* < 0.05 and *p* < 0.001, respectively).

Autonomic nervous system (ANS) parameters were analyzed as post- to pre-30k-RUN ratios to assess differences between MAG and CTRL garment conditions. Paired *t*-tests revealed significantly greater increases in the MAG condition compared to CTRL for RMSSD (*t*_(14)_ = −2.525, *p* = 0.030, *d* = −0.761, 95% confidence interval [CI], −1.423 to −0.071), HF power (*t*_(14)_ = −2.310, *p* = 0.036, *d* = −0.598, 95% CI, −1.140 to −0.037), and HF/(LF + HF) ratio(*t*_(14)_ = −2.600, *p* = 0.021, *d* = −0.671, 95% CI, −1.224 to −0.099). Conversely, the LF/HF ratio exhibited a significantly greater increase in the CTRL condition compared to MAG (*t*_(14)_ = 4.213, *p* < 0.001, *d* = 1.088, 95% CI, 0.431 to 1.720) (Figure 3). Other ANS parameters similarly showed a tendency toward parasympathetic dominance and were listed in Supplementary Figure S1.

### 3.3. Perceived Recovery and Deep Sleep Score

**Figure 4:**
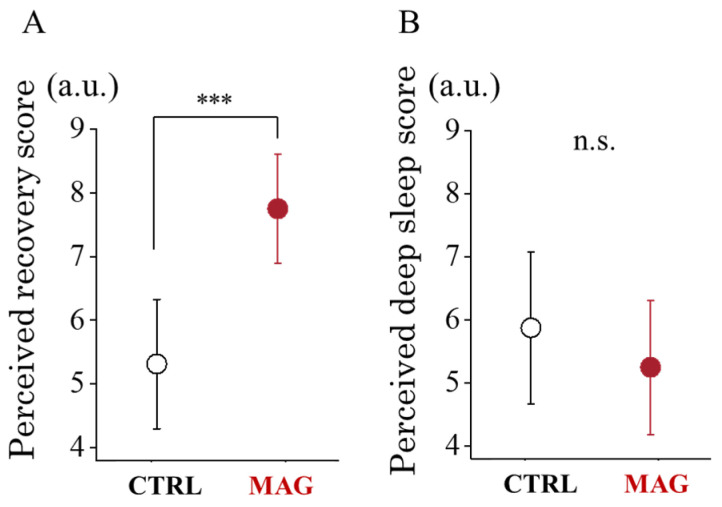
Perceived recovery (**A**) and deep sleep (**B**) scores following 30k-RUN in CTRL and MAG conditions. *** shows a significant difference (*p* < 0.001) between CTRL and MAG conditions.

Following the 30k-RUN, participants in MAG reported significantly higher perceived recovery scores compared to those in CTRL (*t*_(14)_ = −6.197, *p* < 0.001, *d* = −1.597, 95% CI, −2.359 to −0.811). However, no significant difference was observed in deep sleep scores between the MAG and CTRL conditions (*t*_(14)_ = 1.790, *p* = 0.095, *d* = −0.462, 95% CI, −0.079 to 0.988) (Figure 4).

## 4. Discussion

This study aims to investigate the effects of magnetic garments on sleep quality in a specific group of male athletes. After a 30k-RUN, CTRL did not show an increase in parasympathetic dominance or improvements in sleep quality. On the other hand, MAG, when compared to CTRL, experienced enhanced parasympathetic dominance and improved sleep quality. These findings suggest that using magnetic garments may help male long-distance runners recover from fatigue and improve their sleep quality, which could have significant practical implications for their intense training and competitive performance.

### 4.1. Different Responses of Autonomic Status After 30k-RUN Between CTRL and MAG

The quality of sleep plays a significant role in the physical recovery process after intense training [6]. Previous studies have shown that high-intensity exercises can lead to increased sympathetic nervous system activity [19,20] and decreased sleep quality [21,22,23], which can negatively impact performance. Our findings support these conclusions, as we observed that CTRL did not lead to parasympathetic dominance or changes in sleep quality after the 30k-RUN. This suggests that the autonomic nervous system and sleep quality may not respond well to high-intensity endurance exercise. Conversely, it has been reported that extended sleep beyond normal duration had positive effects on endurance performance for triathlon athletes [24]. In our study, wearing magnetic garments led to increased parasympathetic activity and improved sleep quality after the 30k-RUN. Therefore, our results suggest that deliberate strategies to improve autonomic nervous system function and sleep quality are important for recovery from fatigue and exercise-induced damage after high-intensity endurance exercise. Our findings indicate that interventions involving magnetic garments could be an effective approach in this context.

### 4.2. Effects of Magnetic Intervention on Endurance Athletes

The effects of magnetic approaches on physiological processes have been studied in both animal models and non-athlete human populations. Studies have shown that exposure to static magnetic fields can improve blood flow [25,26] and sleep quality [27], potentially affecting the autonomic nervous system. In the present study focusing on a specific athletic population, wearing magnetic garments increased the duration of deep sleep after the 30k-RUN and promoted dominance of the parasympathetic nervous system compared to CTRL. These findings suggest that improved blood flow induced by magnetic field exposure could influence autonomic nervous system function and sleep quality, potentially aiding athletes’ recovery after intense training and competitions. While this study provides evidence for the potential benefits of magnetic garments on sleep quality and autonomic function in endurance athletes, further research is needed to understand the underlying physiological mechanisms. Future investigations should explore the relationship between magnetic field strength, exposure duration, and their effects on sleep and recovery parameters.

### 4.3. Practical Implications and Potential Mechanisms of Magnetic Wear

The present study suggests that wearing magnetic garments may have an impact on the autonomic nervous system and sleep quality of male long-distance runners. However, the specific mechanisms behind this effect are not yet fully understood. While participants reported improved recovery, there was no significant change in the perceived deep sleep score. Since sleep duration was controlled, the enhanced sleep quality may have increased the likelihood of participants falling asleep. Coaches and athletes should consider incorporating this approach into their recovery strategies, especially before important competitions or during intensive training. The authors have proposed potential mechanisms, such as the influence of magnetic fields on bioelectric currents [25], biological membranes [28], and circadian rhythms [29]. However, the exact mechanisms are still unclear. Further research is needed to clarify the specific physiological pathways through which magnetic interventions may impact athletes’ autonomic nervous system and sleep quality.

### 4.4. Methodological Limitations

Several methodological considerations warrant discussion. Researchers and clinicians are increasingly adopting consumer wristbands as tools for measuring outcomes in sleep studies [30,31,32]. While wristband-type devices may be less accurate in measuring sleep quality compared to traditional polysomnography (PSG) [33,34], recent studies have shown good agreement between Fitbit devices and PSG for various sleep parameters [15,16,17]. These devices significantly reduce the time and financial burden associated with collecting longitudinal sleep data and provide rich information that was previously difficult to obtain outside of sleep laboratories or clinics [35,36,37]. However, this limitation may affect the interpretation of results, suggesting that future studies should include or validate findings with more precise measurement tools. Secondly, both the existing literature and the present study primarily focus on short-term magnetic interventions. This design was chosen to align with the athletes’ training camp schedules. It needs to be clarified whether the short-term effects of this study are beneficial in the long term. Future research is needed to understand the long-term effectiveness of magnetic approaches, especially in the context of athlete recovery. Thirdly, our sample size of 15 male collegiate distance runners was relatively small, potentially limiting the generalizability of our results. The sample size was limited by the logistical challenges of conducting a controlled study with elite athletes during their training camp. However, our use of a randomized, double-blind, crossover design helps to mitigate some of the limitations of the small sample size. Future studies should address these limitations by including a more diverse participant pool, encompassing females and athletes of various ages and competitive levels. Fourthly, our study employed a single magnetic field strength, which, indeed, limited conclusions regarding optimal field strength. This highlights the need for dose-response studies to determine the most effective magnetic field strength for athlete recovery. Lastly, the subjective questionnaires may be influenced by various psychological factors. Therefore, future studies should incorporate additional objective measures to complement these subjective assessments.

## 5. Conclusions

After completing the 30 km run, participants who did not receive any intervention did not show improvements in their autonomic nervous system function or sleep quality that would help them recover from exercise-induced damage or fatigue. In contrast, those who wore magnetic garments experienced longer deep sleep and a stronger parasympathetic nervous system response compared to CTRL. These results indicate that wearing magnetic garments may be a practical way to facilitate recovery in male long-distance runners after intense training.

## Data Availability

The original contributions presented in this study are included in the article/Supplementary Materials; further inquiries can be directed to the corresponding authors.

## Supplementary Materials

The following supporting information can be downloaded at https://www.mdpi.com/article/10.3390/s24216820/s1, Figure S1. Relative changes in additional ANS parameters following 30k-RUN in CTRL and MAG conditions.
