# Supplementary material for: Magnetic Garments Promote Parasympathetic Dominance and Improve Sleep Quality in Male Long-Distance Runners Following a 30 km Run
Source: Sensors (Basel). 2024 Oct 23;24(21):6820. doi: 10.3390/s24216820 (PMC11548770; doi:10.3390/s24216820)
Supplement: Supplementary file 1 [file sensors-24-06820-s001.zip › sensors-3226991-supplementary.pdf]

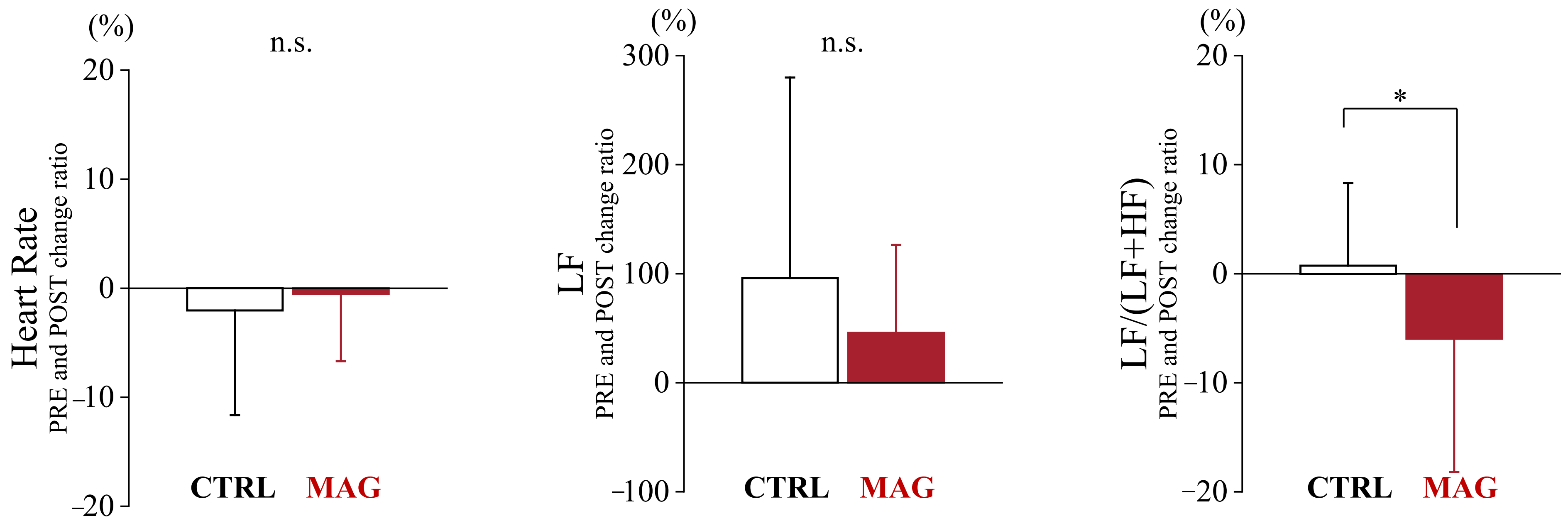

Supplementary Figure S1. Relative changes in additional ANS parameters following 30k-RUN in CTRL and MAG conditions.

n.s. show no significant difference between the CTRL and MAG condition for Heart rate ($t_{(14)}$ = −0.409, p = 0.691, d = −0.123, 95% CI, −0.714 to 0.473) and LF power ($t_{(14)}$ = −1.961, p = 0.078, d = −0.591, 95% CI, −1.223 to 0.065), however, * show significant difference CTRL and MAG conditions for LF/(LF+HF) ($t_{(14)}$ = 2.604, p = 0.026, d = 0.785, 95% CI, 0.089 to 1.452)
